# Th17-mediated antitumor immunity in patient-derived organoid and autologous immune cell cocultures predicts response to immunotherapy in head and neck cancer

**DOI:** 10.1016/j.iotech.2026.101598

**Published:** 2026-06-06

**Authors:** P. Meißner, P. Kaps, T. Momper, A. Wolff, I. Raabe, D.F. Strueder, U. Langenkamp, J. Brandstetter, C. Junghanss, A.S. Becker, A. Zimpfer, C. Maletzki

**Affiliations:** 1 Department of Internal Medicine―Clinic and Polyclinic for Hematology, Hemostaseology, Oncology, Stem Cell Therapy and Palliative Medicine, Rostock University Medical Center, Rostock, Germany; 2 Department of Otorhinolaryngology, Head and Neck Surgery “Otto Koerner,” Rostock University Medical Center, Rostock, Germany; 3 Institute of Pathology, Rostock University Medical Center, Rostock, Germany; 4 Department of Medical Laboratory Diagnostics, Saarland University Medical Center, Homburg, Germany

**Keywords:** antigen-specific T cells, cytotoxicity, T cell exhaustion, T cell stimulation, tumor architecture

## Abstract

**Background:**

Immune checkpoint inhibitor (ICI) therapy improves survival in head and neck squamous cell carcinoma (HNSCC), yet only some patients benefit, highlighting the need for early identification of responders and functional evaluation of tumor–immune interactions. We developed an autologous coculture platform combining patient-derived organoids (PDOs) with peripheral blood mononuclear cells (PBMCs) to assess immune responses and correlate preclinical findings with clinical outcomes.

**Material and methods:**

PDOs from HNSCC specimens were expanded and cocultured with PBMCs, followed by treatment with ICIs targeting programmed cell death protein 1 (PD-1), lymphocyte-activation gene 3 (LAG-3), or their combination.

**Results:**

PBMCs were enriched for CD4^+^ and CD8^+^ T cells (∼90%) and CD16^+^CD56^+^ natural killer cells, with baseline exhaustion (LAG-3^+^, CTLA-4^+^). T cell–mediated tumor killing occurred in 44% of cases and was accompanied by Th1/Th17 cytokine signatures. ICI treatment modestly shifted T-cell phenotypes, reducing exhaustion (PD-1) and enriching cytolytic subsets (CD4^+^IFN-γ^+^, CD8^+^TOX^+^). Interleukin (IL)-17A emerged as a relevant cytokine, correlating with FasL secretion and suggesting activation of the Fas/FasL axis. High circulating IL-17A levels in treatment-naïve HNSCC patients were associated with improved survival, whereas other cytokines showed no prognostic value. In a noninterventional observational trial, increases in IL-17A positively correlated with favorable ICI responses, indicating its potential as a biomarker. PDO viability decreased in response to ICIs in a patient-specific manner, consistent with cytokine signatures and clinical outcomes.

**Conclusion:**

Collectively, this autologous PDO–PBMC coculture platform, combined with longitudinal cytokine profiling, captures patient-specific tumor–immune interactions and interpatient heterogeneity, providing a framework for real-time response prediction, biomarker discovery, and personalized immunotherapy in HNSCC.

## Highlights

• Patient-derived organoid models can be established from fresh and biobanked tumor tissue.
• Patient-derived organoid–PBMC cocultures capture individual tumor–immune interactions and predict ICI response.
• IL-17A may contribute to Th17-mediated antitumor immunity and could serve as prognostic and predictive biomarker in HNSCC.
• ICI therapy promotes cytolytic T-cell response in some cases and appears to engage the Fas/FasL axis within tumors.

## Graphical abstract

**Figure:**
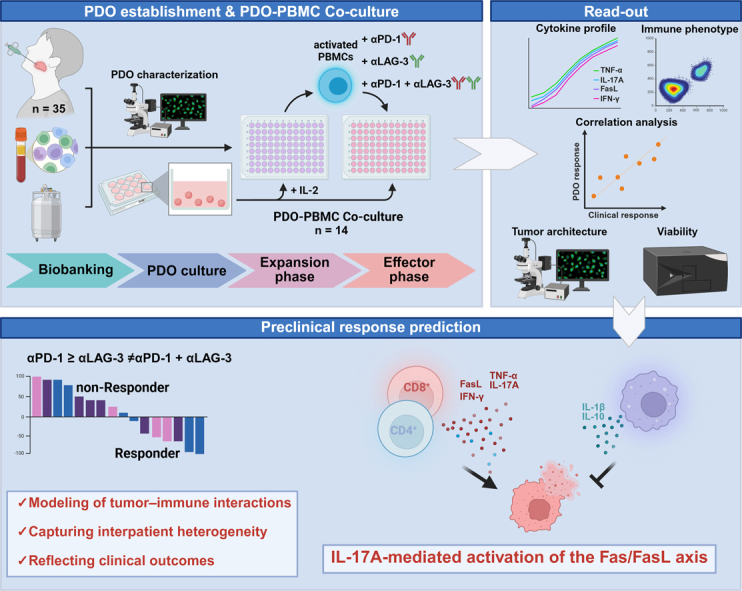

## Introduction

Head and neck squamous cell carcinoma (HNSCC) arises from malignant transformation of epithelial cells, causing ∼770 000 new cases and 385 000 deaths worldwide annually.^1^ Despite multimodal therapy, prognosis remains poor, particularly in advanced disease.

Immune checkpoint inhibition (ICI) is increasingly integrated into HNSCC management, exemplified by the recent approval of perioperative pembrolizumab for resectable disease (KEYNOTE-689).^2^ However, clinical benefit remains limited: programmed death-ligand 1 (PD-L1) combined-positive score assessment is the only approved predictive biomarker, and major pathological responses occur in a minority of patients, with objective response rates of 15%-20% in recurrent/metastatic disease.^3^^,^^4^ Early-phase studies of dual checkpoint blockade [programmed cell death protein 1 (PD-1) with CTLA-4/lymphocyte-activation gene 3 (LAG-3) inhibition] have shown preliminary activity and mechanistic insights.^5^, ^6^, ^7^ Translational models that faithfully recapitulate tumor–immune interactions are therefore needed to guide patient-tailored treatment strategies.^3^

Patient-derived organoids (PDOs) have emerged as a versatile *ex vivo* platform, with studies demonstrating their capacity to reflect treatment responses to chemo- and radiotherapy in HNSCC.^8^, ^9^, ^10^, ^11^ However, most established PDO protocols rely on enzymatic dissociation into single cells followed by extracellular matrix embedding, thereby disrupting the native tumor microenvironment and limiting the capacity to investigate ICI efficacy.^8^^,^^12^^,^^13^

To address these limitations, matrix-free or ‘en bloc’ organoid or explant approaches that preserve stromal and immune architecture have been proposed.^14^, ^15^, ^16^ Building on this concept, coculture with autologous peripheral blood mononuclear cells (PBMCs) may enable functional investigation of systemic tumor–immune interactions, identify patient-specific resistance mechanisms, and immunotherapeutic modulation in an *ex vivo* setting.^17^^,^^18^

We present a streamlined approach to generate matrix-free HNSCC PDOs from both fresh and biobanked tumor specimens, coupled with autologous T-cell cocultures. This platform enables real-time functional assessment of ICI activity, providing a translational model to study patient-specific tumor–immune interactions and guide precision immunotherapy strategies.

## Methods

### Patients and ethical approval

Ethical approval was obtained from the local ethics committee (approval no.: A-2022-0064). Written informed consent was obtained from all participants before study inclusion. Patients with histologically proven HNSCC were included. Tumor tissue was either collected during diagnostic biopsy or surgery, before systemic therapy. Frozen vital tumor tissue [in freezing medium, i.e. fetal calf serum (FCS) + 10% dimethyl sulfoxide (DMSO)] from the Rostock-HNSCC-Biobank^19^ was used from 14 patients. Autologous blood samples were collected in EDTA vials and processed immediately using ficoll density gradient centrifugation (1140 *g*, 15 min, no brake). Additionally, allogeneic blood samples from unrelated healthy donors (EV A2024-0112) were used. PBMCs were harvested from all blood samples, counted, and vitally frozen in liquid nitrogen (10 Mio. PBMC/vial in freezing medium). Further processing and handling of PBMCs are given in the supplementary information ‘priming and expansion phase,’ available at https://doi.org/10.1016/j.iotech.2026.101598).

### PDO establishment

PDOs were established from 38 individual treatment-naïve HNSCC patients. Tissue was either taken fresh from surgery/biopsy or from our local biobank (vitally frozen in FCS + 10% DMSO). Briefly, tumor tissue was washed three times with ice-cold phosphate-buffered saline (PAN-Biotech, Aidenbach, Germany), mechanically dissociated using a McIlwain Tissue Chopper (Cavey Laboratory Engineering, Surrey, UK), followed by washes and red blood cell lysis. Tumor pieces were cultured in PDO medium (12-well suspension plates). HNSCC basal medium consisted of DMEM/F12 (PAN-Biotech) supplemented with 1× penicillin-streptomycin (PAN-Biotech), 1× GlutaMAX™ Supplement (ThermoFisher Scientific, Waltham, MA), 10 mM HEPES, and 5 μg/ml gentamicin (both from Gibco™, ThermoFisher Scientific). PDO medium additionally contained 100 ng/ml Noggin, 100 ng/ml R-Spondin 1 (both PeproTech), 1× N2 Supplement (Gibco™, ThermoFisher Scientific), 1× NCS21 neuronal supplement, serum-free (Capricorn Scientific, Ebersdorfgrund, Germany), 50 ng/ml epidermal growth factor (ImmunoTools, Friesoythe, Germany), 1 mM *N*-acetyl-l-Cystein (Sigma-Aldrich, St. Louis, MO), 10 μM Y-27632 (Selleck Chemicals, Houston, TX), 1× penicillin/streptomycin, 1.25 μg/ml Amphotericin B (Gibco™, ThermoFisher Scientific), and 5 μg/ml gentamicin. Plates were incubated at 37 °C and 5% CO_2_ on an orbital shaker (230 rpm). Every 3-5 days, PDOs were resuspended or manually dissected into 0.5-1 mm fragments, followed by washing and resuspension in new medium. Viability was assessed on day 7 using Calcein AM (1:2000 dilution, 4 mM stock, 20 min, 37°C), followed by microscopic imaging and fluorescence quantification. As a control, non-Calcein AM-stained PDOs were imaged and background fluorescence intensity was measured from each PDO. For quantification, background fluorescence intensity was calculated from the signal after Calcein AM staining. Additionally, before immunofluorescence staining or coculture experiments, PDOs were either gently resuspended or mechanically fragmented into 0.5-1.0 mm pieces using a spring-loaded tissue dissector.

### Histologic and immunofluorescence characterization of PDOs

PDOs were fixed in 4% paraformaldehyde (PFA, 30 min), incubated in 30% sucrose solution overnight, embedded in tissue freezing medium, snap-frozen, and sectioned (4 μm). After blocking with 2% bovine serum albumin (1 h), sections were incubated overnight with antibodies (1:20 dilution): Alexa Fluor 594 anti-pan-Cytokeratin (sc-8018, Santa Cruz Biotechnology, Dallas, TX), Alexa Fluor 647 anti-Ki-67 (Thermo Fisher Scientific), and Alexa Fluor 488 anti-CD326 (EpCAM) (9C4, BioLegend). Nuclei were counterstained with ROTI Mount FluorCare DAPI (Carl Roth, Karlsruhe, Germany), followed by imaging on a fluorescence microscope (Zeiss Axiovert.A1, Jena, Germany).

Additionally, the original tumor tissue was compared with the models (at the time of primary cultivation and during further passages) in terms of tumor cell morphology (cell size, growth pattern, degree of differentiation including keratinization), stromal desmoplasia, and inflammation using a four-point scale. This comparison was done by a trained pathologist.

### Two-phase PDO–PBMC coculture assay

We adapted the protocol from Cattaneo et al.^20^: (i) priming and expansion phase—a 96-well U-bottom plates were precoated with 5 μg/ml anti-CD3/CD28 (both Miltenyi Biotec, Bergisch Gladbach, Germany) and stored overnight at 4°C. T-cell RPMI-1640 medium (PAN-Biotech) supplemented with 10% male human AB serum (PAN-Biotech), 1× penicillin/streptomycin, and 1× GlutaMAX was used. Cryopreserved PBMCs were thawed carefully in T-cell thawing medium (human serum was replaced with FCS), incubated with DNAse (37°C, 15 min), resuspended at 2 × 10^6^ cells/ml in T-cell culture medium (+ 150U/ml IL-2, Proleukin, Iovance Biotherapeutics), and incubated overnight (37 °C). PDOs were prestimulated with 200 ng/ml IFN-γ (ImmunoTools) overnight, stained with Calcein AM, and resuspended in T-cell culture medium. Preactivated PBMCs were washed, adjusted to 1-5 × 10^5^ cells in human serum-containing medium (+ 300U/ml IL-2). PDOs and PBMCs were cocultured at a ratio of 1 PDO to 1-5 × 10^5^ PBMCs (+ 150U/ml IL-2). After 7 days of coculture, PDOs and PBMCs were pooled and counted. (ii) Effector phase with immune checkpoint blockade—the pooled PDO–PBMC mixture was washed, cocultured with new PDOs into anti-CD3/CD28 precoated 96-well U-bottom plates and incubated under the following experimental conditions using technical triplicates: PDOs ± PBMCs, + anti-human leukocyte antigen (HLA)-ABC Clone W6/32 (50 μg/ml, ImmunoTools), + anti-HLA-DR/DP/DQ Clone Tü39 (10 μg/ml, BioLegend), + anti-HLA-ABC/anti-HLA-DR/DP/DQ (50/10 μg/ml, respectively), + nivolumab (40 μg/ml, Opdivo), + pembrolizumab (40 μg/ml, Keytruda, both provided by the pharmacy of Rostock University Medical Center), + relatlimab (40 μg/ml, MedChemExpress), and + nivolumab + relatlimab (40 μg/ml each). For each condition, 5 × 10^5^ prestimulated, T cell–enriched PBMCs were added per PDO. On day 3 of this coculture setup, T cell–mediated killing was analyzed *via* Calcein AM measured on a Tecan Plate Reader Infinite 200 Pro (Tecan Group AG, Ex/Em: 485 nm/535 nm). PDO viability was assessed by measuring fluorescence intensity at day 3 relative to day 0, using the same measurement settings to ensure comparability. A threshold of ≥20% viability reduction was applied, as this cutoff exceeds assay-inherent variability (which is usually ±10%-15%) to capture biologically meaningful responses in such heterogeneous 3D cultures.

### Spectral flow cytometry of immune phenotypes and cytokine profiles

Before cell harvest, Brefeldin A (BioLegend, 1:1000, 4 h) was added. FVD eFluor450 (BioLegend, 1:250) was used for live/dead staining. True-Stain Monocyte Blocker™ (BioLegend) was added before ex/in staining. The detailed protocol is listed in the supplement. Fifty thousand events were measured on a spectral flow cytometer. Additionally, supernatants were analyzed by the LEGENDplex™ Human CD8/NK Panel (13-plex) and the LEGENDplex™ Human Inflammation Panel 1 (13-plex) (both from BioLegend) according to the manufacturer’s instructions. Plasma samples from treatment-naïve HNSCC patients (*n* = 76) and HNSCC patients receiving palliative ICI treatment [*n* = 5, anti-PD1 antibody: 2× nivolumab, 3× pembrolizumab, before and after three cycles, as part of the BIOTRUST-ONC-study (EV A2024-0112)] were additionally included, and plasma cytokine levels were measured using the LEGENDplex™ Human Inflammation Panel. All measurements were carried out on a spectral flow cytometer (3L-Cytek™ Aurora). Data were analyzed using SpectroFlo™ Version 3.2.1 and FlowJo™ Version 10.6.1, respectively The BioLegend LEGENDplex™ Data Analysis Software Suite was used for statistical analysis.

### Statistics

Statistical analysis and data visualization were carried out using Prism GraphPad 10.6.0 (GraphPad Software, San Diego, CA; RRID:SCR_002798). Each data set was tested for normality with the Shapiro-Wilk test. Data are presented as scatter plots, with each point representing an individual case or measurement. For normally distributed data, mean values are shown; for nonnormal distributions, median values are reported. Survival analyses were carried out using Kaplan–Meier curves, and differences between groups were assessed with the log-rank (Mantel–Cox) test. The criterion for significance was set to *P* < 0.05. Correlation analyses were carried out for selected markers based on data characteristics and biological relevance, using nonparametric Spearman correlation. In selected analyses, coefficient of determination (*R*^2^) values were calculated to quantify the proportion of variance explained by the model.

## Results

### Establishment of matrix-free PDOs from fresh and biobanked HNSCC

**Figure 1:**
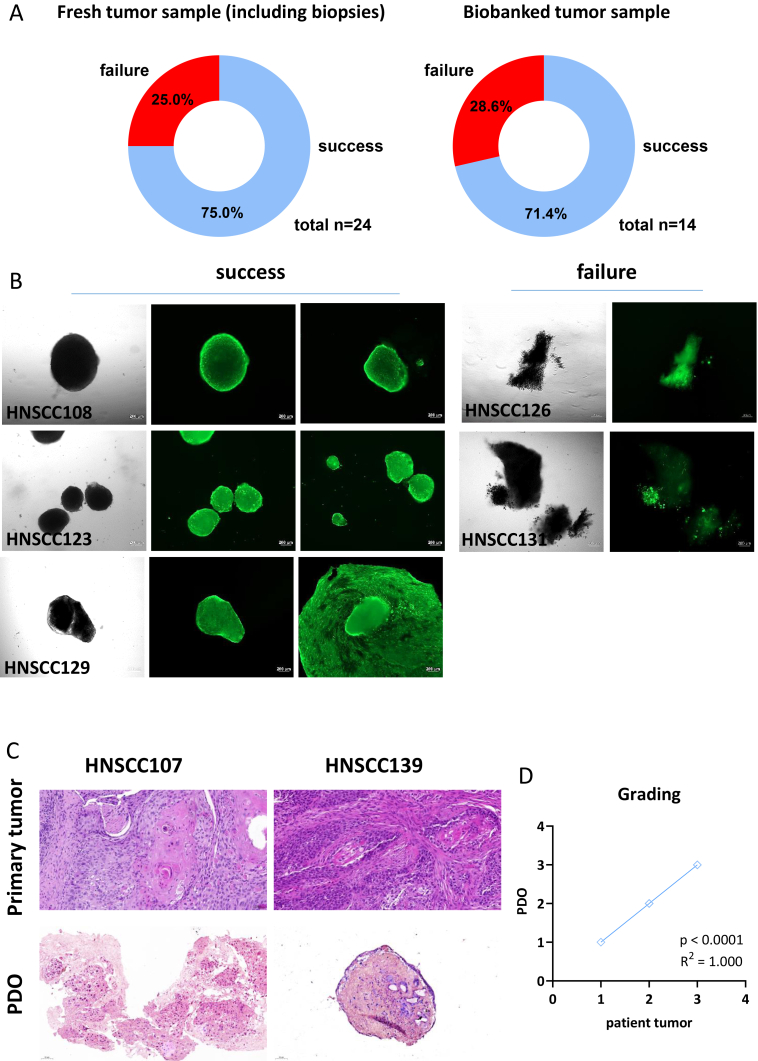
**Establishment of patient-derived organoids (PDO) from HNSCC.** (A) The success rate of PDO establishment from either fresh (*n* = 24) or biobanked (*n* = 14) tumor samples was quantified based on morphology changes and positive Calcein AM staining. (B) PDOs were imaged *via* light and fluorescence microscopy for Calcein AM intensity. Success of the PDO establishment (left) was accompanied by round morphology and a positive Calcein AM staining, whereas failed PDOs (right) were associated with an irregular shape and the absence of viability. Scale bar = 200 μm. (C, D) Morphology of tissue sections and matched PDOs was analyzed via HE histology to show their architectural similarity (*n* = 12 matched cases). Scale bar = 50 μm. Tumor grading was carried out by a pathologist. Nonlinear regression was applied, and the calculated *R*^2^ value is shown in the graph. *P* < 0.0001. HE, Hematoxylin and Eosin; HNSCC, head and neck squamous cell carcinoma.

To preserve the native tumor architecture, we developed a matrix-free culture method for generating PDOs from HNSCC tumor samples (Figure 1), adapted from Ref. 15. Thirty-eight tumor specimens from 35 patients were included (Table 1). In two cases, both primary tumors and synchronous lymph node metastases were obtained.

**Table 1:** Clinical characteristics of the PDO patient cohort

Baseline clinical characteristics	Total (*n* = 35)
Age, years (median, range)	63 (46-88)
Sex, *n* (%)	
Female	6 (17.1)
Male	29 (82.8)
Tumor stage, number (%)	
T1	3 (8.6)
T2	9 (25.7)
T3	11 (31.4)
T4	11 (31.4)
CUP	1 (2.9)
HPV status, number (%)^a^	
Positive	7 (20.0)
Negative	28 (80.0)
Current or former smoker, number (%)	
Yes	27 (77.1)
No	8 (22.9)
Alcohol consumption, number (%)	
Yes	26 (74.3)
No	9 (25.7)
Anatomical site, number (%)	
Oral cavity	7 (18.4)
Oropharynx	17 (44.7)
Larynx	5 (13.2)
Hypopharynx	3 (7.9)
Lymph node	6 (15.8)
Tissue source, number (%)	
Primary tumor	29 (76.3)
Metastasis	6 (15.8)
Relapse	3 (7.9)
Sample type, number (%)	
Fresh	24 (63.2)
Biobanked	14 (36.8)
Collection method, number (%)	
Biopsy	16 (42.1)
Surgical specimen	22 (57.9)
Time of ischemia, number (%)	
≤1 h	27 (71.0)
>1 h	11 (28.9)
Sample processing, number (%)	
Microdissector	10 (26.3)
Tissue chopper	28 (73.7)
PDO establishment, number (%)	
Success	28 (73.7)
Failure	10 (26.3)
PDO establishment conditions, number (%)	
≤ 1 h time of ischemia	18 (66.7)
> 1 h time of ischemia	10 (90.9)
Microdissector	8 (80.0)
Tissue chopper	20 (71.4)

PDO, patient-derived organoid.

a Human papillomavirus (HPV) status was determined by local testing for participants with oropharyngeal cancer according to p16 immunohistochemical analysis.

PDOs were successfully established from both fresh (63.2%) and biobanked (36.8%) samples, with overall success rates of 75.0% and 71.4%, respectively (Figure 1A). After 1-2 weeks, viable PDOs acquired a rounded morphology (Figure 1B, left), as confirmed *via* Calcein AM fluorescence. Occasional fibroblastic outgrowth, which is typical of single-cell–derived cultures in classical 2D-culture models, required plate transfer. Failed PDOs showed fragment disintegration, a lack of rounded structures, and a low Calcein AM signal (Figure 1B, right). Histopathological staining of primary tumors and matched PDOs revealed structural concordance (Figure 1C and Supplementary Figure S1, available at https://doi.org/10.1016/j.iotech.2026.101598) and faithfully recapitulated key morphological features (Figure 1D). No difference in tumor grade was observed between the primary tumor and the organoid (*R*^2^ = 1.000). Tumor cells were predominantly localized at the organoid periphery, likely reflecting nutrient limitation in the core. Although PDOs did not significantly increase in size, they maintained a stable tumor-like architecture for more than 6 weeks. Prolonged *in vitro* culture led to a decrease in immune cell infiltration in PDOs compared with their matched primaries (Figure 1D).

**Figure 2:**
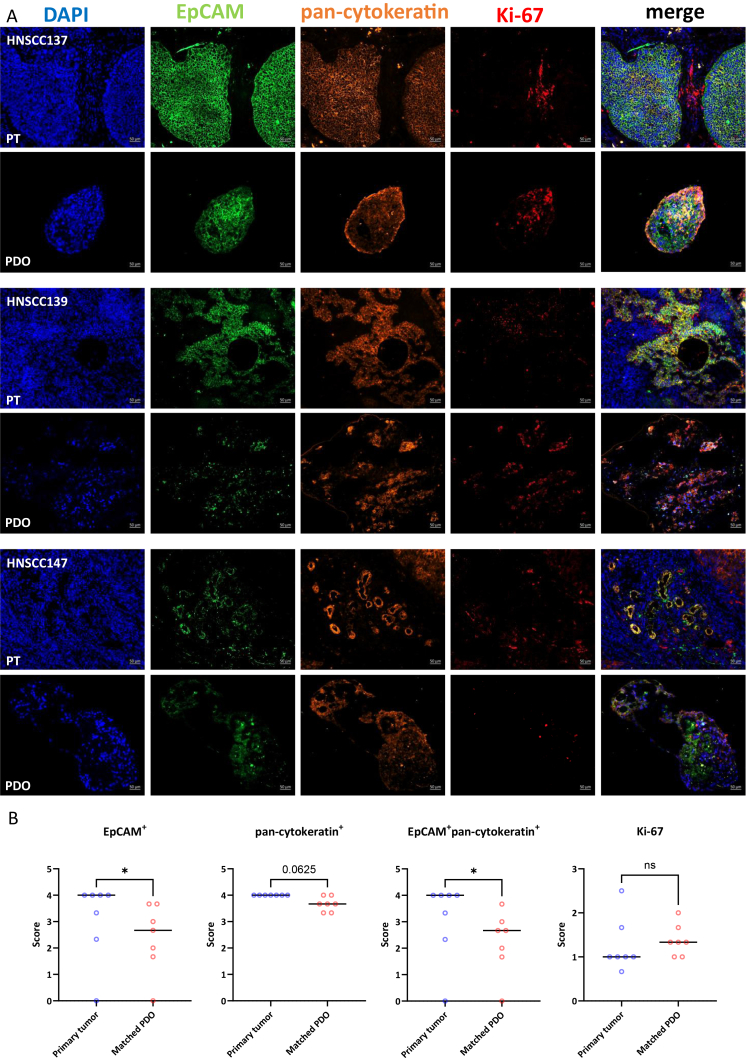
**Preservation of key histological features in the PDO model.** (A) Immunofluorescence staining of tumor tissue and their corresponding PDOs from three different samples is shown as representative images. The samples were cryosectioned, stained for the key HNSCC markers EpCAM and pan-cytokeratin as well as the proliferation marker Ki-67, and imaged using a fluorescence microscope. Scale bar = 50 μm. (B) Target protein abundance was quantified by individually scoring each marker for at least three images per case. Only matched primary tumors and PDOs were used for the quantification. *n* = 7, median. Wilcoxon test, *∗P* < 0.05. HNSCC, head and neck squamous cell carcinoma; PDO, patient-derived organoid; PT, primary tumor.

The presence of key HNSCC markers was confirmed by immunofluorescence in PDOs (Figure 2). Despite a modest reduction in EpCAM^+^ and EpCAM^+^/pan-cytokeratin^+^ cells, both epithelial markers, highly abundant in primary tumors, were preserved in PDOs. Ki-67 staining indicated moderate proliferative activity in both tumors and PDOs (Figure 2B).

Overall, PDOs recapitulate the histoarchitectural organization of their corresponding primary tumor and retain clinically relevant intra- and intertumoral heterogeneity, supporting their use as a physiologically faithful platform for preclinical therapeutic screening.

### Autologous PDO coculture promotes stronger T-cell expansion than allogeneic conditions

PDO–PBMC cocultures were established in either autologous or allogeneic conditions, depending on patient blood availability, and to assess the robustness of the model in the absence of matched patient blood. PDO viability was quantified by fluorescence-based read-outs, whereas T-cell phenotyping and cytokine secretion profiling were done using flow cytometry (*n* = 15 cases).

**Figure 3:**
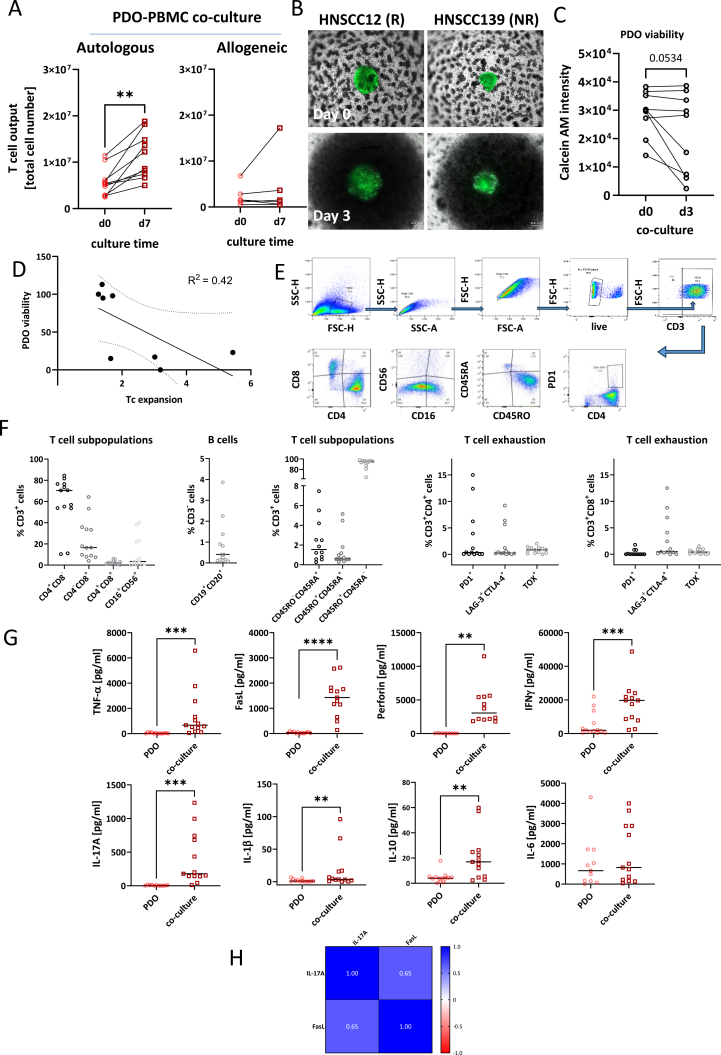
**T-cell activity and subpopulation dynamics in PDO-immune cell coculture.** (A) PDOs were cocultured with either autologous or allogeneic (healthy donor-derived) peripheral blood mononuclear cells (PBMCs). Viable PBMCs were counted before coculture at day 0 and after coculture at day 7. *n* = 10 autologous cases, *n* = 6 allogeneic cases; symbols and lines show individual cell numbers. Paired *t*-test, *∗∗P* < 0.01. (B) Tumor cell killing was examined microscopically at day 0 and day 3 of coculture. PDO viability was visualized by Calcein AM staining. (C) The fluorescence signal of viable tumor cells was measured before coculture at day 0 and at day 3 after coculture using a microplate reader. *n* = 11, symbols and lines show individual values of the PDO cases. Wilcoxon test. (D) The association between PDO viability and T-cell expansion is shown. *n* = 8. Nonlinear regression was applied, and the calculated *R*^2^ value is shown in the graph. (E) Gating strategy and exemplary flow plots for key markers are displayed. (F) Flow cytometry phenotyping of PBMCs to identify distinct immune cell subpopulations in the autologous coculture setting. Shown are the percentage of CD3^+^ T cells positive for the indicated markers, and percentages of CD3^+^CD4^+^ or CD3^+^CD8^+^ T cells expressing exhaustion markers. *n* = 13, median. (G) The concentration of secreted cyto- and chemokines in the supernatant of the coculture model was evaluated by a multiplex bead-based immunoassay. n = 13, mean. Wilcoxon test or paired *t*-test, *∗∗P* < 0.01*, ∗∗∗P* < 0.001, and *∗∗∗∗P* < 0.0001. (F, G) Measurements were done after 7 days of coculture. (H) The association between FasL and IL17A cytokine levels in cell-free supernatants from PBMC–PDO cocultures was calculated and displayed as a correlation matrix (*Spearman* correlation). *n* = 12. *P* < 0.05. HNSCC, head and neck squamous cell carcinoma; NR, nonresponder; PDO, patient-derived organoid; R, responder.

Prestimulated autologous T cells showed significantly higher expansion when cocultured with matched PDOs compared with allogeneic T cells (average expansion rate: 2.3 ± 1.3 versus 1.3 ± 0.8, *P* < 0.05, Figure 3A). A reduction in PDO viability was observed in 44.4% of cases (*P = 0.05*), consistent with T cell–mediated cytotoxicity, whereas the remaining 55.6% showed viability comparable to baseline (Figure 3B, C). T-cell responses were highly patient-specific. Still, the extent of PDO viability decline correlated positively with T-cell expansion rate (*Spearman r = 0.69*, Figure 3D).

Subsequent T-cell phenotyping revealed additional differences between the two coculture settings (representative flow plots and the gating hierarchy are shown in Figure 3E and Supplementary Figure S2C, available at https://doi.org/10.1016/j.iotech.2026.101598). These findings should be interpreted cautiously, as the analysis included only two healthy donors. Nevertheless, autologous patient PBMCs tended to contain a higher proportion of CD4^+^ T helper cells, accompanied by lower numbers of cytotoxic CD8^+^ T and natural killer (NK) cells (Figure 3F). The distribution of naïve (CD45RO^-^CD45RA^+^), transitory (CD45RO^+^CD45RA^+^), and memory (CD45RO^+^CD45RA^-^) T cells was comparable across both conditions. Expression of T-cell exhaustion-associated markers varied between patients, with some showing elevated levels and others being within normal levels (Figure 3F, Supplementary Figure S2). Generally, PD-1, LAG-3, and CTLA-4 were slightly higher expressed on CD4^+^ than on CD8^+^ T cells, likely reflecting the higher abundance of T helper cells in general. TOX, a transcription factor strongly associated with T-cell exhaustion, was detectable at very low frequency in the peripheral blood; when present, TOX^+^ cells were predominantly CD4^+^ T cells. HLA blockade resulted in a marked reduction in detectable T cells (both CD4^+^ and CD8^+^ subpopulations, as well as natural killer T cells), consistent with impaired TCR–major histocompatibility complex (MHC) interactions (Supplementary Figure S5, available at https://doi.org/10.1016/j.iotech.2026.101598).

Cytokine profiling of autologous cocultures revealed significantly elevated levels of the proinflammatory cytokine TNF-α along with cytotoxic mediators FasL, perforin, and IFN-γ, and Th17-cytokine IL-17A (Figure 3G, H). FasL and IL17A levels were positively correlated (*Spearman r = 0.65*), suggesting a functional link between these two factors. This cytokine pattern is consistent with robust T-cell activation, although the concurrent increase in IL-1β and IL-10 indicates engagement of innate immune pathways that may impose negative feedback on cytotoxic effector responses. TNF-α and IFN-γ may synergistically enhance antigen presentation and promote FasL/perforin-mediated tumor killing, supported by IL-17A, whereas IL-1β and IL-10 likely counteract this response, reflecting negative regulatory feedback within the coculture system. Although not statistically significant, elevated IL-6 levels further point to a complex interplay between T-cell activation and counter-regulatory immunosuppression that may ultimately limit cytotoxic potency.

### Individual sensitivity of PDOs to PD-1 and LAG-3 blockade

**Figure 4:**
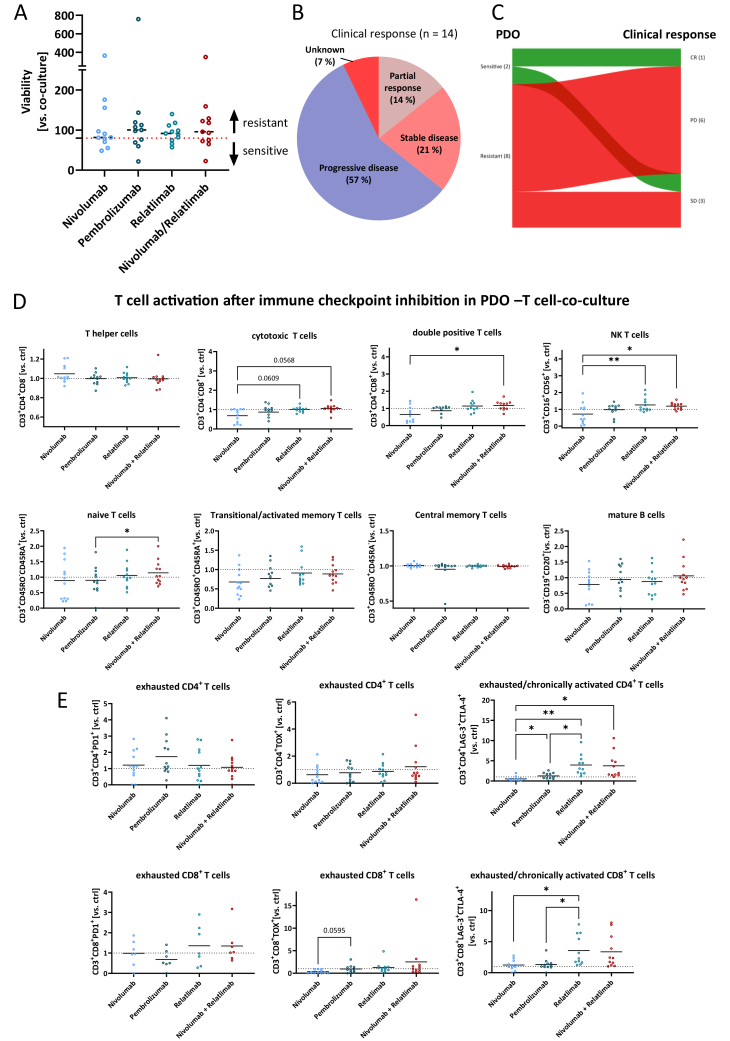
**Immune checkpoint inhibition in PDO–T-cell coculture and correlation with patient response.** (A) Autologous PDO–T-cell cocultures were treated with the immune checkpoint inhibitors (ICIs) nivolumab, pembrolizumab, relatlimab, or the simultaneous combination of nivolumab and relatlimab (40 μg/ml each) for 3 days. Thereafter, PDO viability was assessed by Calcein AM assay, measuring fluorescence intensity. Viability was then calculated in relation to baseline fluorescence intensity. For response evaluation, a binary scoring system was applied, defined by a ≥20% change from baseline, indicated in the graph by the red dotted line. *n* = 11, median. (B) Distribution of clinical response of HNSCC patients (*n* = 14) treated with ICI is displayed. (C) Sankey plot to show the correlation of the individual patient’s clinical response with the corresponding PDO. *n* = 7. (D, E) The PDO–T-cell coculture was supplemented with the ICIs nivolumab, pembrolizumab, relatlimab, or the simultaneous combination of nivolumab and relatlimab (40 μg/ml each) for 3 days. Thereafter, T cells were harvested, counted, and stained. The percentage of CD3^+^ T-cell subpopulations (D) and the exhausted CD3^+^CD4^+^ or CD3^+^CD8^+^ T cells (E) were characterized using flow cytometry. *n* = 12, mean. One-way ANOVA (Tukey’s multiple comparisons test), *∗P* < 0.05*, ∗∗P* < 0.01. ANOVA, analysis of variance; HNSCC, head and neck squamous cell carcinoma; ICI, immune checkpoint inhibitor; NK, natural killer; PBMC, peripheral blood mononuclear cell; PD, progressive disease; PDO, patient-derived organoid; PR, partial response; SD, stable.

Next, we evaluated the impact of ICI treatment in the coculture system to model clinical application. Clinically approved anti-PD-1 inhibitors nivolumab and pembrolizumab, and the anti-LAG-3 inhibitor relatlimab, tested either as monotherapy or in combination with nivolumab, were applied across 12 individual patient cases. PDO viability was finally evaluable from 11 cases (Figure 4A), typically based on three technical replicates per condition (i.e. three individual PDOs, Supplementary Figure S2B, available at https://doi.org/10.1016/j.iotech.2026.101598). In one case (HNSCC128), PDOs derived from both the primary tumor and a matched synchronous metastasis were cocultured with autologous PBMCs, enabling direct comparison of ICI responses across tumor sites.

Responses were heterogeneous and highly patient-specific, with only minor differences observed between PD-1 inhibitors and the LAG-3 inhibitor. No consistent response pattern emerged, as variability was evident both across PDO samples and treatment regimens (Supplementary Figure S2B, available at https://doi.org/10.1016/j.iotech.2026.101598). Therefore, a binary scoring system was applied to classify samples as responders or nonresponders, defined by a ≥20% change from baseline. Using this criterion, 45.5% of samples were classified as sensitive to PD-1 blockade with nivolumab, whereas 36.4% and 27.3% were sensitive to pembrolizumab or relatlimab, respectively. Combination treatment did not overcome resistance; PDO viability remained similar to that seen with single-agent checkpoint blockade. Two cases demonstrated broad ICI sensitivity, showing 30%-80% viability reduction across all agents tested. In the matched primary–metastasis pair, the primary tumor was resistant to all ICIs that phenotype persisted in the synchronous metastasis (Supplementary Figure S3A, available at https://doi.org/10.1016/j.iotech.2026.101598), suggesting outgrowth of an intrinsically resistant subpopulation during metastatic progression.

When these findings were compared with clinical outcomes from HNSCC patients receiving palliative anti-PD-1 treatment, i.e. either pembrolizumab or nivolumab (*n* = 14, Supplementary Table S1, available at https://doi.org/10.1016/j.iotech.2026.101598), 57% of patients showed progressive disease (Figure 4B). Objective response rates were <40% (stable disease/partial response). To further evaluate the translational relevance of our platform, we directly compared *ex vivo* findings with clinical responses. Matched data from 10 patients receiving identical ICI regimens (i.e. either nivolumab or pembrolizumab) enabled a correlative analysis. Notably, the autologous PDO–PBMC coculture system reproduced the differential response patterns observed in the clinic, as illustrated by the side-by-side comparison in Figure 4C. Despite the limited sample size, the concordance between preclinical and clinical outcomes supports the ability of this model to capture patient-specific immune responsiveness. Although the concordance between *ex vivo* and in-patient outcomes is encouraging, definitive conclusions about predictive value will require validation in larger, prospectively enrolled cohorts.

As a starting point, we next investigated the relationship between individual cases and ICI sensitivity. Correlation analysis revealed a positive association between responses to nivolumab/pembrolizumab and relatlimab (*Spearman r = 0.81* and *0.73*, respectively, Supplementary Figure S3B, available at https://doi.org/10.1016/j.iotech.2026.101598), indicating that cases sensitive to PD-1 blockade tend to also respond to LAG-3 inhibition, and *vice versa*. Consistent with this, 3/4 PDOs sensitive to nivolumab and 2/4 PDOs sensitive to pembrolizumab also responded to relatlimab (Supplementary Figure S3C, available at https://doi.org/10.1016/j.iotech.2026.101598). Here again, correlations in larger cohorts—ideally embedded within coclinical trial designs—will help to determine the robustness, generalizability, and clinical utility of this approach for anticipating treatment responses and informing individualized immunotherapy strategies in HNSCC.

### LAG-3 blockade triggers an activated, antigen-specific T-cell signature while maintaining features of an exhausted phenotype

**Figure 5:**
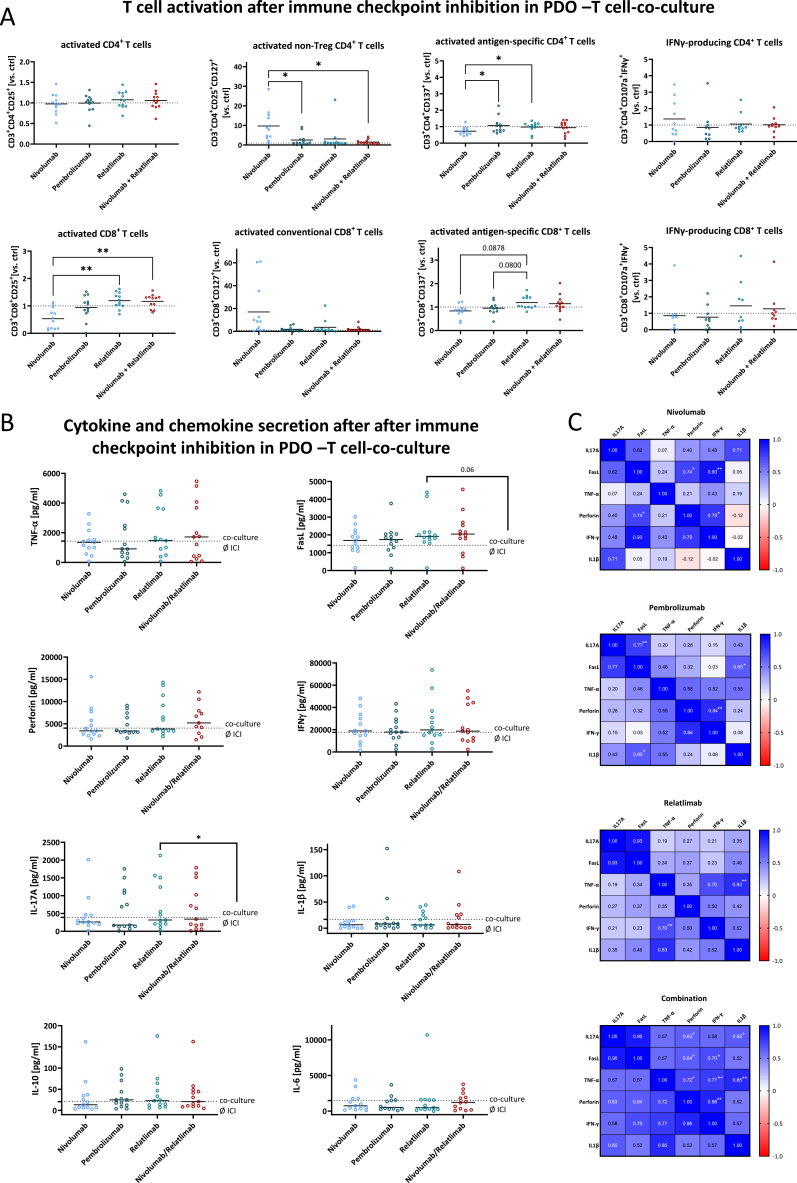
**T-cell activation and cytokine secretion after immune checkpoint inhibition in PDO–T-cell coculture.** (A) Autologous PDO–T-cell cocultures were treated with nivolumab, pembrolizumab, relatlimab, or the combination of nivolumab and relatlimab (40 μg/ml each) for three days. Thereafter, T cells were harvested, counted, and stained. The percentage of activated CD3^+^CD4^+^ or CD3^+^CD8^+^ T-cell populations was quantified after 3 days using flow cytometry. *n* = 12, mean. One-way ANOVA (Tukey’s multiple comparisons test), *∗P* < 0.05*, ∗∗P* < 0.01. (B) Cytokine and chemokine concentrations in coculture supernatants were measured after 3 days of ICI treatment using a multiplex bead-based immunoassay. The dotted line indicates the concentration in untreated cocultures. *n* = 13, median. One-way ANOVA or Friedman-Test (Dunn’s multiple comparisons test), *∗P* < 0.05. (C) Cyto-/chemokines with potential relevance in T cell–driven cytotoxicity and their interconnection within this regulatory network were used for correlation analysis. Therefore, values from cyto-/chemokines following the four ICI treatment regimens were correlated and displayed as a correlation matrix (*Spearman* correlation). *n* = 12. *∗P* < 0.05*, ∗∗P* < 0.01. ANOVA, analysis of variance; ICI, immune checkpoint inhibitor; PDO, patient-derived organoid.

The impact of ICI treatment on T-cell phenotypes was thoroughly assessed after autologous coculture (*n* = 12; Figures 4D, E and 5). Cocultures without ICIs served as controls and were normalized to 1 for graphical comparison. Additional controls using MHC I and II blocking antibodies that were included in the analysis are given in Supplementary Figure S5, available at https://doi.org/10.1016/j.iotech.2026.101598; these data were not considered for direct comparisons with ICIs.

Numbers of CD4^+^ helper and CD8^+^ cytotoxic T cells did not differ significantly between treatment groups. In contrast, nivolumab/relatlimab increased the proportion of CD4^+^CD8^+^ T cells (*P* < 0.05 versus nivolumab). Relatlimab, both as monotherapy and in combination, also elevated NK T cell frequencies (*P* < 0.01 and *P* < 0.05 versus nivolumab, respectively). A similar trend was observed for naïve T cells, which were more abundant after combination ICI treatment. Transitional and memory T-cell subsets, as well as B cell numbers, remained unchanged.

Then, we examined exhaustion markers on CD4^+^ and CD8^+^ T-cell subsets. PD-1 blockade with nivolumab or pembrolizumab only modestly reduced PD-1^+^ and TOX^+^ cells, with a trend toward stronger effects for nivolumab. Relatlimab primarily induced patient-specific responses. In contrast, pronounced differences emerged in LAG-3^+^ and CTLA-4^+^ cell populations, which were significantly upregulated on both CD4^+^ and CD8^+^ T cells in the relatlimab group. In the combination, T-cell exhaustion was confined to CD4^+^ T cells, whereas CD8^+^ expression patterns varied among patients. Because LAG-3 and CTLA-4 can also reflect chronic activation, we assessed activation markers and found that relatlimab activates T cells more effectively than PD-1 blockade (Figure 5). CD8^+^ T-cell activation (CD3^+^CD8^+^CD25^+^) was significantly increased with relatlimab (*P* < 0.01 versus nivolumab) and similarly elevated in the combination (*P* < 0.01 versus nivolumab), underscoring the strong activation potential of anti-LAG-3 therapy. In contrast, nivolumab primarily activated CD4^+^CD127^+^ effector cells, an effect absent in the combination, suggesting antagonistic interactions that may account for the reduced T cell–mediated PDO toxicity observed in coculture. Activated CD127^+^ CD8^+^ T cells remained unchanged across all treatment conditions, pointing to *de novo* T-cell activation rather than expansion of preexisting populations.

Finally, assessment of CD137 revealed significantly higher numbers of activated antigen-specific CD4^+^ T cells in the pembrolizumab and relatlimab groups (*P* < 0.05 versus nivolumab), with comparable increases in the CD8^+^ population. Although not reaching significance, individual patients showed signs of activation (= elevated IFN-γ) and cytotoxic degranulation (= elevated CD107a levels), albeit inconsistently across ICI treatments.

These data highlight patient-specific ICI response patterns, with PD-1 blockade enhancing general antitumor immunity and LAG-3 inhibition primarily driving cytotoxic T-cell activation against autologous HNSCC PDOs.

### Cytokine signatures define patient-specific T-cell activation patterns and highlight IL-17A as a potential key mediator

Cytokine profiles were evaluated after autologous PBMC–PDO coculture in 12 cases, including the matched primary–metastasis pair HNSCC128 (Figure 5). Focusing on cytokines significantly altered by coculture (see Figure 3), proinflammatory mediator secretion was largely preserved under ICI treatment. PD-1 blockade induced modest increases in TNF-α, FasL, perforin, and IFN-γ in most cases, although responses varied substantially between patients, consistent with the heterogeneity observed in T-cell phenotyping.

LAG-3 blockade produced a distinct pattern. This antibody enhanced FasL secretion (*P = 0.06* versus coculture), an effect also observed with the combination. Perforin and IFN-γ levels were likewise elevated, despite considerable interpatient variability.

IL-17A levels, which were already significantly elevated in the cocultures without ICIs (see Figure 3F), increased further in some cases. The highest values were seen following LAG-3 blockade (*P* < 0.05 versus coculture) and in the combination. Because this pattern suggested a potentially important role for this Th17-associated cytokine, we examined IL-17A secretion at the individual patient level. Although limited by the small sample size, an inverse correlation emerged between IL-17A levels and PDO viability: cultures with the highest IL-17A concentrations showed the lowest PDO viability after PD-1 or LAG-3 blockade (Supplementary Figure S3D, available at https://doi.org/10.1016/j.iotech.2026.101598; *P* < 0.05 for nivolumab; *P* < 0.01 for relatlimab). This relationship was not seen for the other cytokines, whose secretion patterns were more variable and appeared largely stochastic.

Levels of IL1β, IL-6, and IL-10 remained comparable to coculture controls, indicating that ICI treatment did not counteract PDO-induced regulatory cytokine responses.

Functional analyses revealed coordinated cytokine behavior, with positive correlations among proinflammatory and antitumoral mediators and negative or absent correlations with IL-1β (Figure 5C). Notably, FasL and IL-17A had a strong positive association across all ICI groups, most pronounced with relatlimab mono- and combination therapy (*Spearman r = 0.93* and *0.90*, respectively). This supports the interpretation that ICIs—albeit to different degrees—activate the Th1/Th17 axis.

Correlating these immune signatures with clinical outcomes (objective response rates) in patients receiving PD-1 blockade (Supplementary Table S1, available at https://doi.org/10.1016/j.iotech.2026.101598) revealed that IL-17A concentrations in coculture supernatants positively associated with treatment efficacy (*Spearman r = 0.42*, Supplementary Figure S3E, available at https://doi.org/10.1016/j.iotech.2026.101598). This relationship exceeded that of PD-L1 CPS (*Spearman r = 0.24*), the currently only approved biomarker for ICI treatment, suggesting that IL-17A may capture aspects of antitumor immunity not reflected by PD-L1 expression alone. Although the cohort size limits definitive biomarker claims, these findings illustrate how this coculture platform can mechanistically dissect immune–tumor interactions and identify cytokine signatures linked to functional antitumor activity. Scaling this approach to larger cohorts could enable systematic discovery of immune activation patterns predictive of ICI responsiveness and help refine biomarker strategies beyond PD-L1 CPS.

### IL-17A may constitute a novel marker for response prediction in HNSCC—two case reports

Clinical response patterns of two individual HNSCC cases treated with ICI were analyzed in detail to mechanistically link in-patient outcomes with *ex vivo* immune behavior. One responder (HNSCC107) and one nonresponder (HNSCC139) were selected for comparative immune profiling (Supplementary Figure S4, available at https://doi.org/10.1016/j.iotech.2026.101598). Both tumors were PD-L1–positive, with CPS >10 (HNSCC107) and CPS >60 (HNSCC139). CT imaging before and after therapy (Supplementary Figure S4A, available at https://doi.org/10.1016/j.iotech.2026.101598) confirmed a marked reduction in tumor burden in HNSCC107 under nivolumab, whereas HNSCC139 exhibited rapid progression despite pembrolizumab.

These divergent clinical outcomes were consistent with the preclinical findings in our coculture model: HNSCC107 was identified as ICI-sensitive, characterized by coordinated activation of Th1/Th17-associated effector pathways; coculture with nivolumab induced strong secretion of IL-17A, IFN-γ, and FasL. This cytokine pattern was accompanied by expansion of activated T-cell subsets, including CD4^+^ and CD8^+^CD25^+^CD127^+^ T cells, as well as increased frequencies of CD8^+^CD137^+^ antigen-specific T cells (Supplementary Figure S4B, C, available at https://doi.org/10.1016/j.iotech.2026.101598). Together, these features reflect a robust, polyfunctional antitumor response capable of overcoming tumor-driven immunosuppression.

In contrast, HNSCC139 exhibited a fundamentally different immune landscape. Pembrolizumab exposure failed to induce meaningful increases in proinflammatory cytokines, suggesting an inability to initiate or amplify cytotoxic effector programs. This was evidenced by minimal induction of proinflammatory cytokines, lack of T-cell activation, and slightly higher T-cell exhaustion (TOX^+^). This profile is consistent with a microenvironment in which PD-1 blockade cannot effectively reinvigorate T-cell function, resulting in persistent tumor immune evasion and clinical nonresponse.

These mechanistic distinctions underscore the capacity of the coculture platform to resolve patient-specific immune dynamics and to identify functional signatures that parallel real-world therapeutic outcomes. Scaling this approach across larger cohorts may enable systematic discovery of immune activation patterns and resistance mechanisms that inform biomarker development and guide individualized ICI-based treatment strategies.

### IL17A may constitute a novel prognostic biomarker in HNSCC

**Figure 6:**
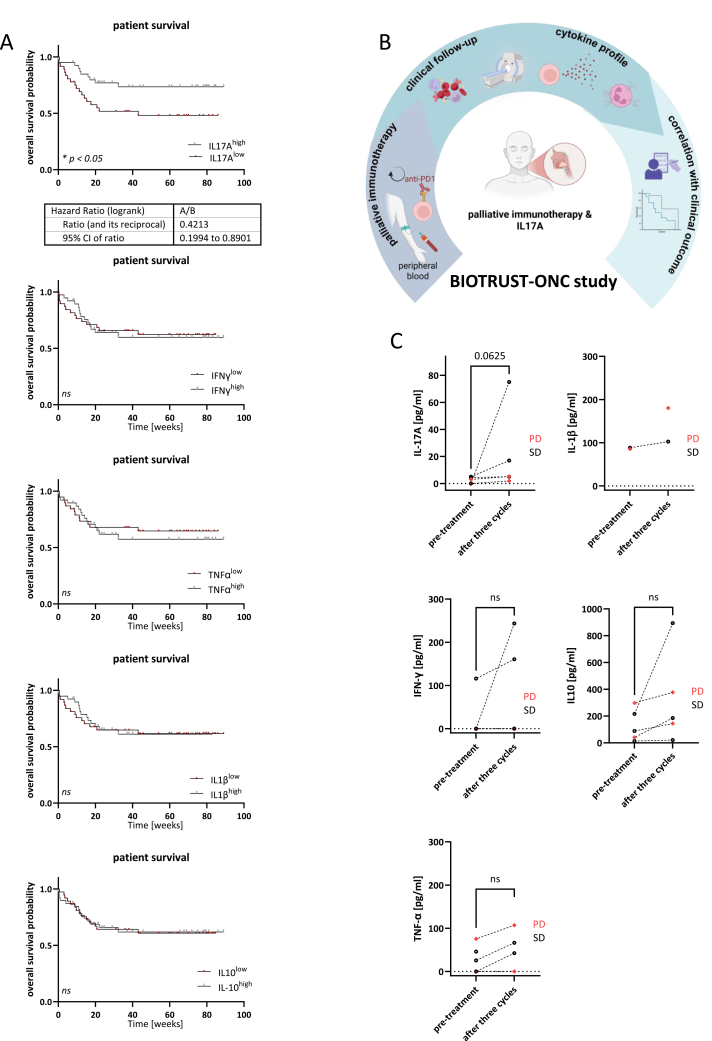
**Prognostic value of plasma cytokines in HNSCC and their association with immunotherapy response.** (A) Treatment-naïve HNSCC patients were divided into cytokine-high and cytokine-low groups based on the median value for each marker. Overall survival was analyzed using Kaplan–Meier estimates, and differences between groups were assessed using the log-rank (Mantel–Cox) test. *P* < 0.05. IL-17A (*n* = 36 high; *n* = 40 low), IFNγ (*n* = 38 high; *n* = 38 low) TNF-α (*n* = 38 high; *n* = 38 low) IL-1β (*n* = 37 high; *n* = 39 low), IL-10 (*n* = 37 high; *n* = 39 low. The hazard ratio is plotted for IL-17A, indicating high versus low. (B) Schematic overview of the ICI workflow with HNSCC patients receiving palliative immunotherapy. Patients were recruited within the BIOTRUST-ONC study, and plasma was obtained longitudinally. (C) Plasma cytokine levels at baseline and after treatment initiation. *n* = 5 HNSCC patients. Each line represents one patient. Different colors indicate clinical response (red, progressive disease; black, stable disease). ICI, immune checkpoint inhibitor; IFN, interferon; HNSCC, head and neck squamous cell carcinoma; PBMC, peripheral blood mononuclear cell; PDO, patient-derived organoid; TNF, tumor necrosis factor.

Finally, we assessed the prognostic significance of IL17A, compared with well-established markers, like IFNγ and TNFα, as well as IL-1β and IL10 (Figure 6A). Stratifying treatment-naïve HNSCC patients (*n* = 76) into high- and low-cytokine groups identified that elevated IL17A emerged as the only cytokine with clear prognostic relevance (*P* < 0.05; HR = 0.4 for IL17A^high^). No comparable prognostic associations were observed for the other cytokines; hence, this selective prognostic signal suggests that IL-17A reflects a biologically meaningful immune state rather than general inflammation.

Cytokine monitoring in five patients undergoing PD-1 blockade (nivolumab, *n* = 2; pembrolizumab, *n* = 3), recruited within the BIOTRUST-ONC-study (*DRKS-ID: DRKS00037366*), further supports this mechanistic link. IL17A levels increased at the on-treatment timepoint (*P = 0.06* versus pre-ICI) and coincided with stable disease, whereas IFNγ, TNFα, IL1β, and IL10 displayed heterogeneous, noninformative patterns.

Together, these data support our concept of an integrated multimodal framework that combines PDO–T-cell cocultures with longitudinal plasma cytokine profiling, as a robust system for response prediction and for dissecting immune–tumor interactions in real time.

## Discussion

Our findings demonstrate that (i) autologous PBMC–PDO cocultures effectively model ICI-mediated antitumor responses in HNSCC and that (ii) the glioblastoma platform^15^ can be adapted to this tumor type with minor modifications.^21^^,^^22^

PDOs combine practical advantages—rapid establishment, scalability, and fewer ethical constraints than traditional *in vivo* xenografts—with clinical alignment further supported by the recent removal of mandatory preclinical animal testing in the United States^23^ and by the emerging evidence regarding their predictive value in precision oncology.^24^^,^^25^ Importantly, we demonstrate that PDOs can be generated from both fresh and cryopreserved HNSCC samples, enhancing logistical flexibility. In our study, PDO establishment rates exceeded 70%, consistent with prior reports^8^^,^^12^^,^^26^^,^^27^ and providing a robust and scalable platform for future coclinical trials.

Treatment responses to ICIs have been infrequently explored in PDO-based systems. Our adaptation of an established preclinical immunotherapy screening platform^28^ enabled both autologous and allogeneic PBMC–PDO cocultures capable of modeling patient-specific immune–tumor interactions. Approximately half of the cases exhibited measurable tumor killing activity, reflecting the interpatient variability in T-cell priming, antigen specificity, and effector competence characteristic of HNSCC. A recent study likewise showed enhanced T cell–mediated cytotoxicity in autologous PBMC–PDO cocultures, evidenced by increased amounts of CD107a and CD137 in CD8^+^ T cells.^29^ Mechanistically, IFN-γ and IL-2 were sufficient to enhance antigen presentation and drive robust T-cell expansion *in vitro*, indicating that cytokine-driven conditioning of PDOs can upregulate immunogenic pathways and support the proliferation of tumor-reactive T-cell clones without the need for additional therapeutic agents. In our study, autologous cocultures with IFN-γ-prestimulated PDOs similarly exhibited a markedly higher expansion rate than allogeneic immune cells, consistent with the presence of circulating tumor-reactive T-cell clones capable of proliferating upon recognition of patient-specific antigens. Functionally, PDOs induced IFN-γ and TNF-α secretion and promoted T-cell activation, differentiation, and cytotoxic effector function. This response was augmented by IL-17A, a Th17-associated cytokine classically linked to inflammation, but increasingly recognized for its context-dependent immunomodulatory roles.^30^ Although IL-17A secretion varied substantially between patients, we observed a strong concordance between IL-17A levels and T cell–mediated killing. No such relationship was observed for the other cytokines (such as TNF-α, IFN-γ, IL-1β, or IL-10), suggesting a mechanistic axis in which IL-17A enhances cytotoxicity. All cytokines examined principally converged on activation of NF-κB, JAK/STAT3, and PI3K/AKT signaling pathways in HNSCC cells, indicating that they share core downstream inflammatory and survival-associated signaling nodes despite their distinct upstream triggers. A direct consequence of this coordinated signaling network is enhanced chemokine production, upregulation of antigen-presentation machinery, and increased susceptibility of tumor cells to immune-mediated killing.^31^^,^^32^ Elevated IL-17A, in particular, likely reflects activation of a Th17-skewed inflammatory program that amplifies cytotoxic effector pathways within the tumor–immune synapse. IL-17A can promote recruitment and activation of neutrophils and myeloid cells, enhance local production of proinflammatory cytokines, and potentiate IFN-γ–driven cytotoxicity. In our system, high IL-17A levels coincided with increased FasL and perforin release and reduced PDO viability, consistent with a coordinated Th1/Th17 effector response capable of overcoming tumor-mediated suppression. The absence of similar correlations for the other cytokines underscores the specificity of this axis and suggests that IL-17A may serve as a surrogate marker for a broader cytotoxic activation state rather than acting as a direct effector alone.

ICI releases the brakes on T cells, and optimally, induces T-cell activation, alleviates T-cell exhaustion, and promotes proinflammatory cytokine secretion, thereby improving antigen presentation and cytotoxicity *via* FasL/perforin-mediated killing pathways. In our system, T-cell activation was moderate, yet direct comparison of ICIs revealed that PD-1 blockade elicited stronger immune-stimulatory effects than LAG-3 blockade. Although interpatient variability was high, responses to PD-1 and LAG-3 inhibition were positively associated, suggesting shared mechanisms of sensitivity or resistance. Consistent with this, dual blockade did not overcome resistance, indicating that intrinsic ICI responsiveness is likely governed by the presence and functional state of antigen-specific T cells rather than by the specific checkpoint targeted (i.e. PD-1 or LAG-3). This observation contrasts with findings from HNSCC mouse models and a clinical melanoma trial, where dual blockade enhanced CD8^+^ T-cell receptor signaling, altered T-cell differentiation, and improved tumor killing.^33^^,^^34^ Notably, even in the melanoma setting, activated T cells retained features of exhaustion—a pattern that closely mirrors our results, particularly following LAG-3 or dual blockade. Both activated CD4^+^ and CD8^+^ T cells upregulated LAG-3 and CTLA-4, while retaining TOX-positivity, consistent with a population of exhausted or chronically activated cells. These exhausted or chronically activated cells can persist long term in the tumor, enabling them to respond to ICIs despite their limited direct cytotoxic capacity. Instead, they contribute indirectly to antitumor immunity through cytokine secretion and modulation of other immune cell subsets.

The antitumor effects in our study were accompanied by increased IL-6 and IL-10, indicating activation of counter-regulatory pathways. IL-6 can promote neutrophil and myeloid-derived suppressor cell recruitment, supporting tumor survival and angiogenesis, whereas IL-10 dampens T-cell activity and may constrain sustained effector responses. As discussed above, IL-17A also participates in this cytokine network, although its contribution to immunotherapy responses appears highly context-dependent.^35^, ^36^, ^37^, ^38^, ^39^ In melanomas, IL-17A promotes T-cell activation and immune infiltration and has been proposed as a biomarker of ICI response.^40^ In pancreatic cancer, IL-17A can impair cytotoxic cell function while improving cancer vaccine efficacy.^41^ Similar context-dependent effects are reported in colorectal cancer, where IL-17 blockade emerged as a strategy to convert immunologically ‘cold’ tumors into ‘hot’ ones.^42^^,^^43^ In HNSCC, IL17A may exert beneficial effects, with studies reporting associations with humoral immune responses, T-cell activation, and cytokine signaling.^44^ Together, these findings highlight a dynamic interplay between activating and regulatory signals that shapes the magnitude and quality of ICI responses in HNSCC PDO–PBMC cocultures.

Moreover, our data reveal a previously unrecognized role for IL-17A in enhancing T cell–mediated cytotoxicity. This effect appears to be modulated by ICI treatment and may involve FasL, as suggested by the correlation between these mediators. These findings support a model in which ICIs differentially engage the Th1/Th17 axis, with potential clinical implications and a rationale for IL-17A–driven patient stratification. Importantly, high circulating IL-17A levels were associated with improved survival in treatment-naïve HNSCC patients, and IL-17A further increased during ICI therapy. In contrast, other cytokines, including TNF-α, IFN-γ, IL1β, and IL10, neither correlated with survival nor changed in response to treatment. Collectively, these observations reinforce IL-17A as a clinically relevant biomarker with both prognostic and potential predictive value in HNSCC, which should be validated in larger patient cohorts.

In tumors harboring preexisting antigen-specific T cells, LAG-3 or PD-1 blockade enhanced antitumor activity, whereas tumors lacking such specificity failed to mount cytolytic activity, likely reflecting interpatient variability in T-cell priming. These observations reinforce that antigen recognition, T-cell competence, and a permissive microenvironment are the dominant determinants of ICI responsiveness. Although antigen specificity was not assessed at the individual level, our data underscore the context-dependent nature of ICI efficacy and position IL-17A as a potential modulator of T cell–mediated immunity in HNSCC. IL-1β also emerged as a mechanistically relevant cytokine: it showed negative correlations with antitumoral mediators and was associated with IL-17A after nivolumab, suggesting a treatment-specific crosstalk. Its absence in other ICI cohorts indicates that this interaction is context-specific rather than universal.

Several mechanistic and methodological limitations should be acknowledged: (i) Model heterogeneity and standardization: PDOs recapitulate patient-specific tumor states but remain molecularly heterogeneous; further molecular characterization is needed to improve reproducibility and capture the full spectrum of HNSCC biology, including inflammatory and differentiation programs relevant to ICI responsiveness; (ii) Immune cell representation: the coculture system lacks tumor-resident immune populations—particularly tumor-infiltrating lymphocytes and draining-lymph-node–derived T cells—that undergo antigen priming, exhaustion, and spatial conditioning *in vivo*. Their absence limits the ability to model key mechanisms such as neoantigen-driven clonal expansion, spatially restricted cytotoxicity, and suppressive myeloid–T-cell interactions. (iii) Lack of dynamic and transcriptional profiling: live-cell imaging and advanced molecular analyses—such as single-cell RNA-seq or TCR sequencing—would provide deeper insight into T-cell dynamics, synapse formation, clonal expansion, or effector differentiation, all of which are central to understanding how ICIs rewire T-cell behavior. These features are central to understanding how PD-1 and/or LAG-3 blockade modulate T-cell behavior and secretion profiles of IL-17A, IL-1β, TNF-α, and other; (iv) Study design and cohort size: the retrospective design and limited a small cohort constrain mechanistic inference and generalizability. Prospective evaluation, ideally in a perioperative setting, would enable controlled sampling and longitudinal immune monitoring. (v) Patient cohort composition: human papillomavirus (HPV)-positive tumors were underrepresented, reflecting local epidemiology but limiting extrapolation to HPV-associated HNSCC, where antigenic landscapes, cytokine milieus, and T-cell differentiation pathways differ substantially. Prospective clinical trials integrating perioperative PBMC–PDO assays could validate cytokine signatures, including IL-17A and IL-1β, as biomarkers of ICI efficacy and resistance. Finally, preclinical testing of rational combination strategies—such as IL-17A or IL-1β blockade with PD-1 inhibition—may uncover novel therapeutic avenues to counteract tumor-promoting inflammation and potentiate cytotoxic T-cell responses.

### Conclusion

We established an efficient method to generate PDOs, providing a suitable platform for studying immunotherapy response. Examination of tumor–immune dynamics in autologous contexts preserves interpatient heterogeneity, accurately mirroring clinical conditions and facilitating response prediction in coclinical trial frameworks. Collectively, our results underscore the promise of IL-17A–driven personalized immunotherapy approaches in HNSCC.
