# Supplementary material for: Th17-mediated antitumor immunity in patient-derived organoid and autologous immune cell cocultures predicts response to immunotherapy in head and neck cancer
Source: Immunooncol Technol. 2026 Jun 6;30:101598. doi: 10.1016/j.iotech.2026.101598 (PMC13264355; doi:10.1016/j.iotech.2026.101598)
Supplement: Supplementary Figures [file mmc1.pdf]

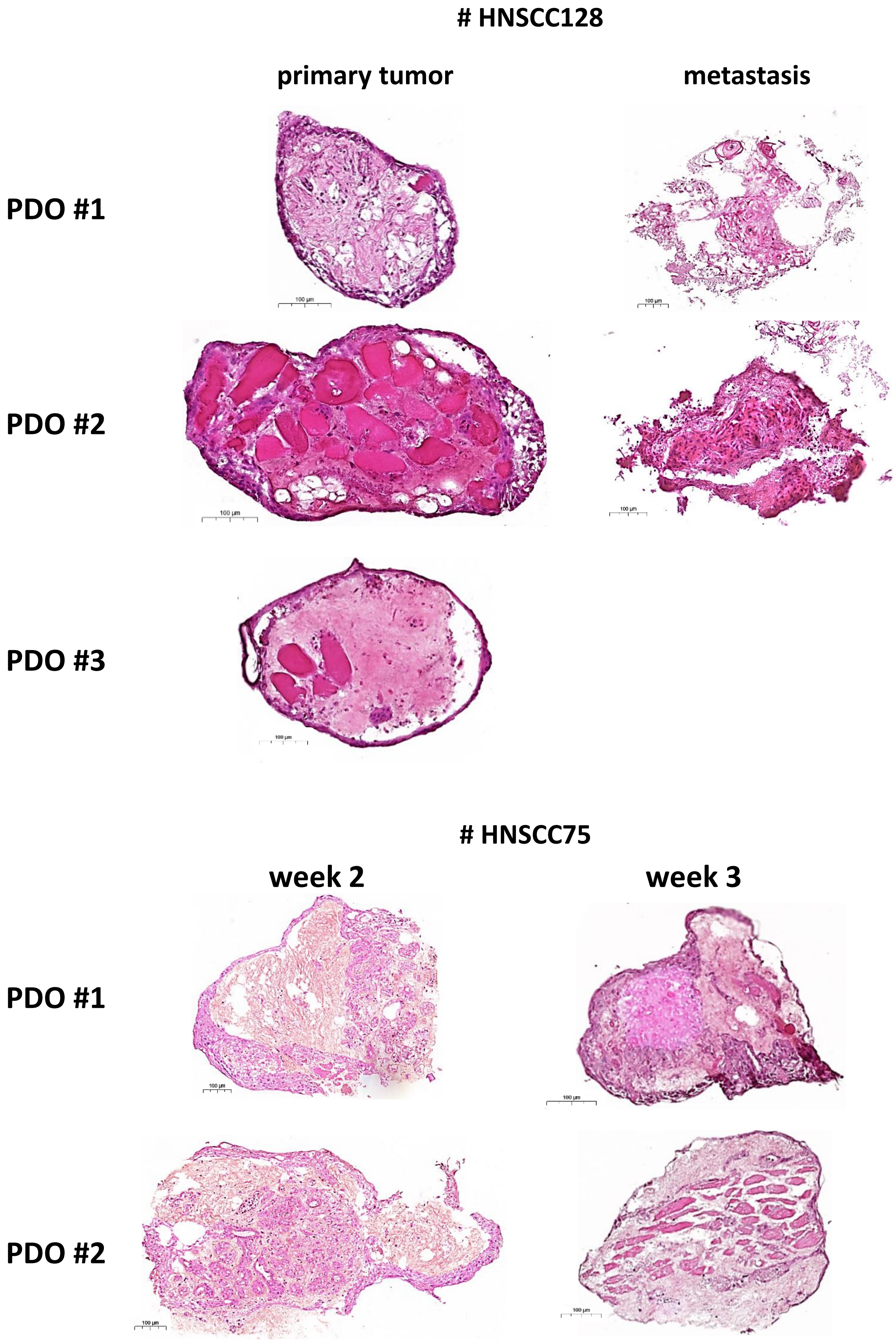

Supplementary Figure 1

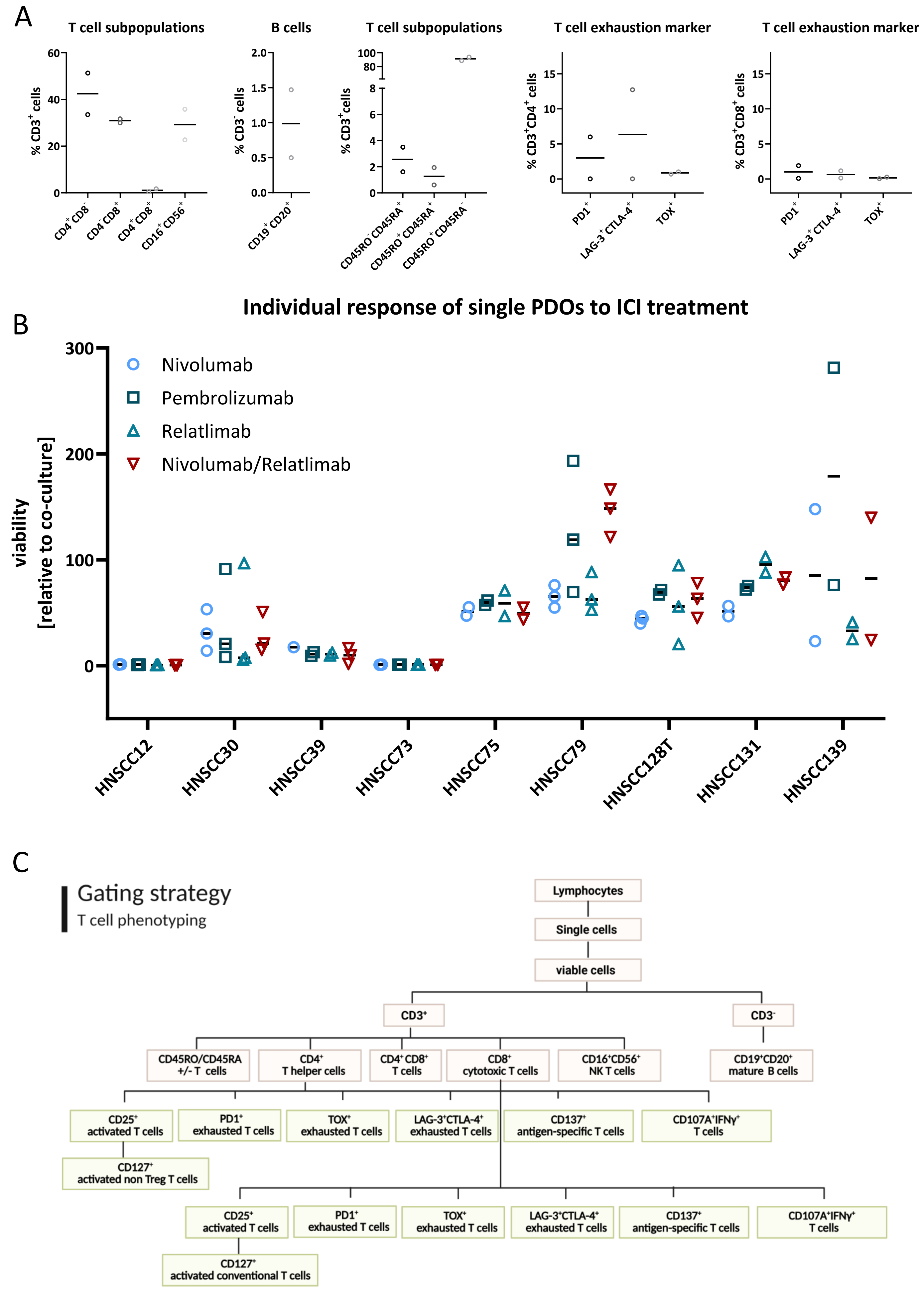

Supplementary Figure 2

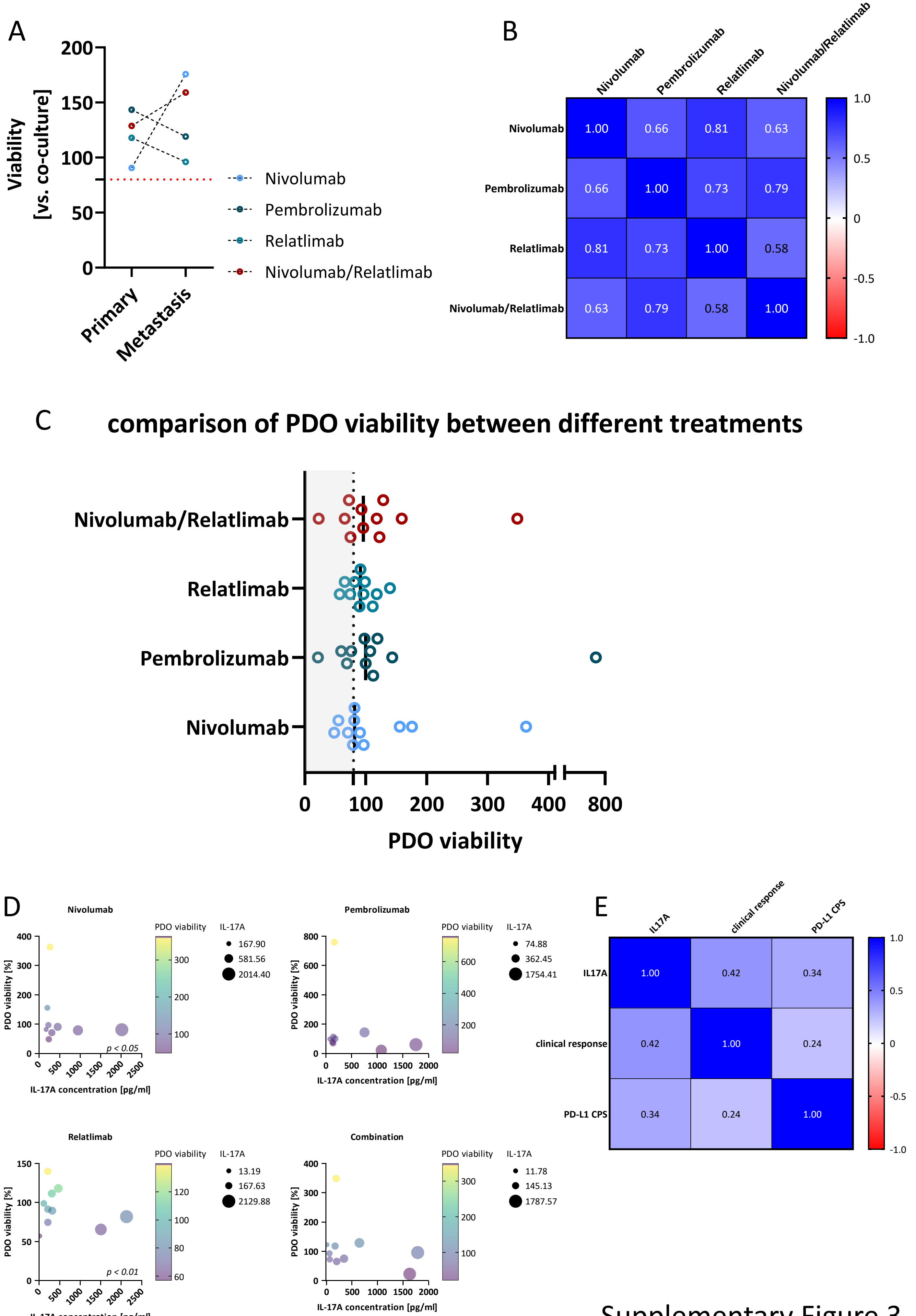

Supplementary Figure 3

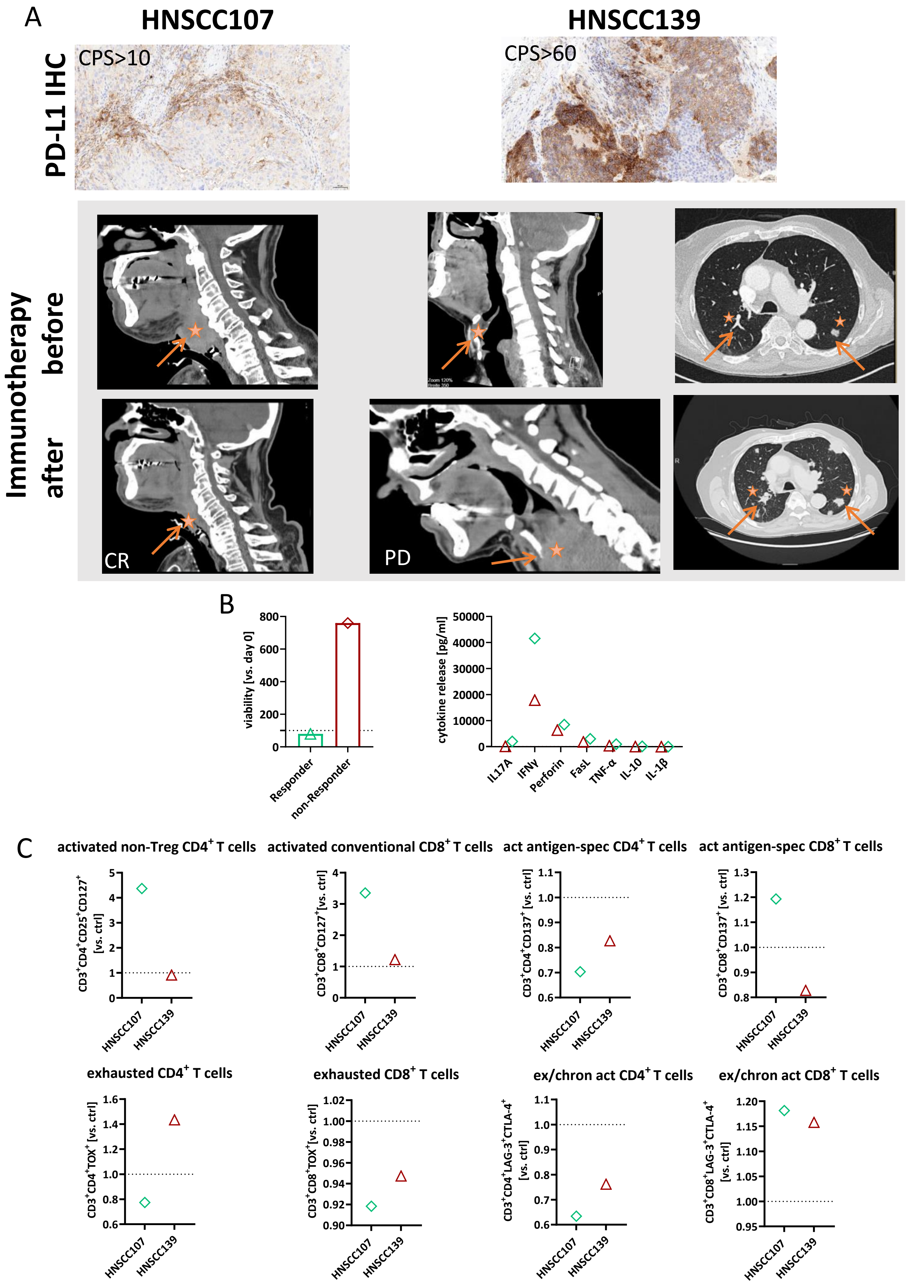

Supplementary Figure 4

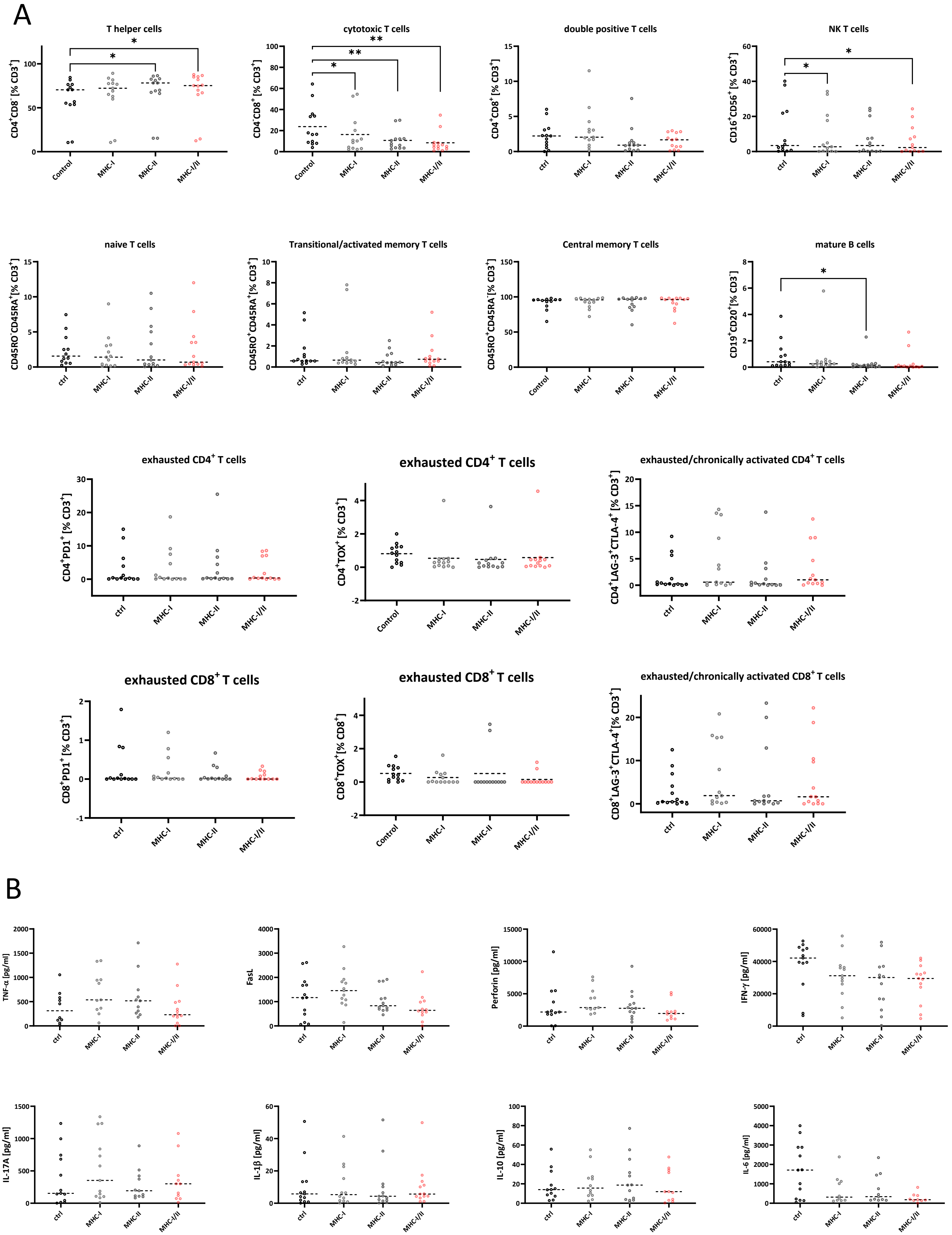

Supplementary Figure 5
