# Supplementary material for: Th17-mediated antitumor immunity in patient-derived organoid and autologous immune cell cocultures predicts response to immunotherapy in head and neck cancer
Source: Immunooncol Technol. 2026 Jun 6;30:101598. doi: 10.1016/j.iotech.2026.101598 (PMC13264355; doi:10.1016/j.iotech.2026.101598)
Supplement: Supplementary Tables [file mmc2.docx]

**Supplementary Table 1. Clinical characteristics of the ICI patient cohort.**

| Baseline Clinical Characteristics | Total (n=14) |
| --- | --- |
| Age, years (median, range) | 62 (51-81) |
| Sex, n (%)  Female  Male | 1 (7.1)  13 (92.9) |
| Tumor stage, number (%)  T1  T2  T3  T4  CUP | 0  4 (28.6)  6 (42.9)  3 (21.4)  1 (7.1) |
| HPV status, number (%)*  positive  negative | 1 (7.1)  13 (92.9) |
| CPS, number (%)  ≥ 10  ≥ 1 < 10  < 1  unknown | 7 (50.0)  4 (28.6)  2 (14.3)  1 (7.1) |
| Current or former smoker, number (%)  yes  no | 8 (57.1)  6 (42.9) |
| Alcohol consumption, number (%)  yes  no | 10 (71.4)  4 (28.6) |
| Clinical response, number (%)  Response  Stable disease  Progressive disease  unknown | 2 (14.3)  3 (21.4)  8 (57.1)  1 (7.1) |
| Anatomical site, number (%)  Oral cavity  Oropharynx  Larynx  Hypopharynx  Lymph node | 4 (28.6)  4 (28.6)  1 (7.1)  2 (14.4)  3 (21.4) |
| Tissue origin, number (%)  Primary tumor  metastasis  relapse | 9 (64.3)  3 (21.4)  2 (14.3) |
| Collection method, number (%)  Biopsy  Surgical specimen | 5 (35.7)  9 (64.3) |
| PDO establishment, number (%)  Success  Failure | 10 (71.4)  4 (28.6) |

*Human papillomavirus (HPV) status was determined by local testing for participants with oropharyngeal cancer according to p16 immunohistochemical analysis.
